# PRELIMINARY YEAR-ONE RESULTS OF A 5-YEAR PROJECT TO DEVELOP AND IMPLEMENT PATIENT- AND FAMILY-CENTERED CARE MODEL

**DOI:** 10.1093/geroni/igz038.1848

**Published:** 2019-11-08

**Authors:** Ya-Mei Chen, Yuchi Young, Dulmaa Munkhtogoo, Ming-Ting Yang, Hsin-Yun Tsai, Nien-Chen Kuo, Tsung-Hsien Yu, Kuo-Piao Chung

**Affiliations:** 1 National Taiwan University, Taipei, Taiwan; 2 SUNY at Albany, Albany, New York, United States; 3 Institute of Health Policy and Management, National Taiwan University, Taipei, Taiwan; 4 Department of Health Care Management, National Taipei University of Nursing and Health Sciences, Taipei, Taiwan

## Abstract

Objective. The overarching goal of this 5-year study is to develop and evaluate an innovative patient- and family-centered care (PFCC) model to address the challenges of quality care and high cost care in Changhua County, Taiwan. The year-1 study examines the baseline differences on selected outcomes between Changhua County and the comparison group (nationally). Methods. Five-year longitudinal study. Participants: stroke patients (n=2,931) from Changhua County Hospitals. Year-1 baseline discharge registry data (2018) for stroke patients obtained from Changhua Health Bureau. Summary statistics and bivariate analysis performed. Providers: care provider [n=28] were interviewed through five focus groups for the support needed to begin implementing PFCC. Results. The year-1 results show the stroke incidence rate was 3.8%. Sixty-three percent of stroke patients were 65+ with an average age of 69, and 60% were male. Compared to the Taiwan national average, the stroke patients in Changhua County had a longer length of stay in acute and postacute care settings (41.1days vs. 29.3 days; p < 0.001), and higher hospitalization cost (US7,815 vs. US$5,905; p<0.001). The focus group data found lack of effective tools and platforms to facilitate the provider-to-provider communication necessary for PFCC. Conclusion. These findings suggest that stroke patients in Changhua County had a longer average length of stay and higher healthcare costs compared to stroke patients nationally. To address these discrepancies, our year-2 program will focus on PFCC intervention program development and pilot testing, which include a Taiwan-specific PFCC tool and platform, followed by three years of intervention implementation and evaluation.

